# Functional Morphology and Ultrastructure of the Peripheral Antennal Sensillar System of *Graphosoma italicum* (Müller, 1766) (Insecta: Hemiptera: Pentatomidae)

**DOI:** 10.3390/insects15070528

**Published:** 2024-07-12

**Authors:** Jolanta Brożek, Izabela Poprawa, Piotr Wegierek, Adam Stroiński

**Affiliations:** 1 Faculty of Natural Science, Institute Biology, Biotechnology and Environmental Protection, University of Silesia in Katowice, 40-007 Katowice, Poland; jolanta.brozek@us.edu.pl (J.B.); izabela.poprawa@us.edu.pl (I.P.); piotr.wegierek@us.edu.pl (P.W.); 2 Polish Academy of Science, Museum and Institute of Zoology of the Polish Academy of Sciences, 00-818 Warsaw, Poland

**Keywords:** morphology, sensilla types, ultrastructure of the dendrites, TEM and SEM techniques

## Abstract

**Simple Summary:**

The study focuses on the morphological characteristics and sensory structures of the antennae of *G*. *italicum*. The results indicate that there is no significant sexual dimorphism in the antennomeres and sensilla equipment. Six main types of sensilla were identified, including basiconic, trichoid, coeloconic, chaetic, campaniform, and peg sensilla. These sensilla were further categorized into subtypes based on their shape, length, and ultrastructure, with evidence supporting their functions as olfactory, thermo–hygroreceptors, and mechanoreceptors. The ultrastructure of the dendritic elements and pore systems of the sensilla, as well as the presence of inflexible/flexible sockets, provided insights into the primary functions of the sensilla. The distribution of sensilla varied across specific antennomeres, with distinct arrangements observed on the scapus, pedicel, basiflagellum, and distiflagellum. The study’s findings provide detailed insights into the morphological and functional diversity of the antennal sensory structures in *G*. *italicum*, contributing to a comprehensive understanding of sensory perception in this species.

**Abstract:**

The antennae of the shield bug *Graphosoma italicum* (Müller, 1766) were examined through scanning and transmission electron microscopy to reveal their general morphology, as well as the antennal sensilla’s distribution, size, and ultrastructure of their dendrites and function. The antennae comprise five antennomeres (one scape, two pedicels, and two flagellomeres). Different lengths of chaetic mechanosensilla (Ch1-Ch4) exist on all antennomeres, and several highly sensitive campaniform sensilla are embedded in the exoskeleton and measure cuticular strain. One pair of peg sensilla, the typical proprioceptive, is only on the proximal edge of the first pedicel and directed to the distal edge of the scapus. The antennal flagellum possesses two subtypes of trichoid and basiconic sensilla, each with one type of coeloconic olfactory sensilla. The distinctive characteristics of *G*. *italicum* are also apparent in two subtypes of coeloconic sensilla embedded in different cavities on both antennomeres of the flagellum, probably with a thermo-hypersensitive function. All studied morphological types of the sensilla and their function were supported by ultrastructural elements. The long and thin trichoid sensilla type 2 (TrS2) with an olfactive function was the most abundant sensilla localized on both flagellomeres. The peripheral antennal sensilla system consists of six main types of sensilla divided into twelve subtypes.

## 1. Introduction

Heteroptera (true bugs) includes more than 42,000 described species worldwide, grouped into seven infraorders. A variable number of superfamilies have been recognized within the infraorder Pentatomomorpha, with five currently accepted: Aradoidea, Coreoidea, Lygaeoidea, Pentatomoidea, and Pyrrhocoroidea [1]. Over 4700 species belong to the Pentatomidae family (stink bugs), and most of them are herbivores with a highly polyphagous nature and the ability to survive unfavorable conditions [2].

*Graphosoma italicum* is a species of red–black shieldbug belonging to the subfamily Podopinae within the family Pentatomidae [3]. The species is regarded as a transpalaearctic element [4], is associated with umbellifers (Apiaceae Lindl.), and is present in Europe and the Middle East [5]. Shieldbugs, in general, are highly distasteful to predators [6] because they can release a repellent secretion, predominantly from their thoracic scent glands when touched. Sillén-Tullberg and Leimar [7] reported that *Graphosoma* is an aposematic and gregarious insect. It would be easy for an avian predator to learn its unpalatability because the bird can encounter another prey item soon after the first.

Chemical communication is a critical element for many insects. The behaviors of *Graphosoma* and other pentatomids or pyrrhocorids predominantly rely on a network of chemical signals during aggregation, complemented by vibration-based signals. The alarm glands produce pheromones such as monoterpenes (especially (E)-2-hexenal), which act as an alarm signal in families such as Pentatomidae, Acanthosomatidae, and Pyrrhocoridae and also serve a defensive role [8]. Gonzaga-Segura et al. [9] and Taszakowski et al. [10] state that the gregarious behavior of these heteropteran bugs may require the same olfactory sensilla to recognize aggregation pheromones in conspecifics of both sexes.

Insect antennae have a wide range of sensilla structures that perform olfactory, tactile, thermo, humidity, and sometimes gustatory functions [11]. Neurons or neuron groups, together with auxiliary cells (thecogen, trichogen, and tormogen) and external cuticular hair-like extensions, form a sensory mini-organ called a sensillum. Axons of the sensilla are collected into bundles, which finally come together in the antennal nerves. The olfactory axons do not have individual glial sheaths, unlike those found around mechanosensory axons, but usually, a larger bundle is enveloped by a standard glial cell [12].

Sensilla are distributed throughout the insect’s body, with a significant number being mechanosensilla [13]; however, chemosensilla, particularly those on the antennae, play the most crucial role in olfaction, making the antennae the primary olfactory peripheral system of insects [14,15,16]. The specific functional composition and arrangement of receptors in these sensilla play critical roles in host recognition, location, mating, aggregation, and other ecological behaviors [17]. Therefore, olfaction is a crucial sensory modality for controlling many aspects of behavior using volatile stimulants [18].

Specific olfactory sensilla in insects show a variety of shapes, including long and short hair-like or plate-like structures, which may have single or double cuticular walls. These sensilla are generally multiporous, and the many small holes penetrating the cuticle provide odor molecules access to the chemosensory neurons [19,20].

Insects have olfactory systems of considerable sensitivity, and many volatile chemicals are perceived by the olfactory receptor neurons (ORNs) inside the antennae. ORNs can sense volatile chemicals with remarkable sensitivity and specificity [21,22,23]. The odorous molecules diffuse through pores in the sensilla walls and are transferred through the sensillum lymph by odorant-binding proteins (OBPs) towards the dendritic processes of the sensory neurons [21,22]. The dominant sensory groups also include various mechanoreceptors with highly sophisticated mechanical properties that have evolved to match insects’ environmental needs. These receptors are responsible for the mechanosensory system, comprised of large, more or less evenly spaced hair-like sensilla, external receptors (campaniform sensilla), and chordotonal organs, which function as a low-frequency extension of the insect’s auditory system [24]. They mediate the detection, localization, and identification of airflow current signals generated by predators, mates, and competitors. They respond to touch and regulate body position [25]. The dominant forms are trichoid and chaetic sensilla, rarely basiconic sensilla, while campaniform sensilla acts as external stretch receptors, sensing deformations of the surrounding cuticle [13,24,25,26,27,28]. Mechanosensilla have no pores and usually contain one neuron sensitive to mechanical stimuli. However, some non-porous sensilla are hygro- or thermosensitive (with different shapes), the most common being the coeloconic sensilla, which includes three neurons [28]. Antennae also bear stimuli of other chemosensory modalities, such as taste and contact-mechanoreception. Both possess one pore (uniporous) but differ in sockets. Gustatory sensilla are usually characterized as basiconic sensilla with inflexible sockets, while contact-mechanoreception sensilla are movable in flexible sockets [8,16].

Representative studies of Pentatomidae have focused mainly on the morphology of the antennal sensilla and their putative functions in *Nezara viridula* (L.) [29,30,31], *Podisus maculiventris* (Say) [32], *Piezodorus guildinii* (Westwood), *Euschistus heros* (Fabricius), *Edessa meditabunda* (Fabricius) [33], *Arma chinensis* Fallou [34], *Eocanthecona furcellata* (Wolff), *Perillus bioculatus* (Fabricius), *Dolycoris indicus* (Stål), *Plautia crossota* (Dallas) [35], and six other species [36]. So far, the ultrastructure and cell organization of the sensilla receptors have been studied in three species: *N*. *viridula* [31], *Halyomorpha halys* (Stål) [37], and *Eurygaster maura* (L.) (Scutelleridae) [38]. The antennae of Pentatomidae can vary significantly when it comes to sensilla sets; sensilla placoidea were found exclusively in *E*. *furcellata*, in contrast to other pentatomid species. Moreover, *D*. *indicus* and *P*. *crossota* showed more sensilla trichodea, basiconica, and chaetica, whereas sensilla coeloconica were restricted to *E*. *furcellata* and *P*. *bioculatus* [35].

According to data from different studies, there are five main types of sensilla in *H*. *halys* [37], six in *P*. *bioculatus*, *D*. *indicus*, and *P*. *crossota* [35], seven in *N*. *viridula* [31], and three types in *N*. *viridula* and *Odontopus nigricornis* Stål (Pyrrhocoridae) [29]. In the Coreidae of *Leptoglossus* species, 14 sensilla types were recognized [10].

These significant differences in sensory endowment in the antennae have many potential values for taxonomic, ecological studies, and behavioral analyses because the antennae of these bugs play an essential role in detecting food, mates, and in the short-range location of conspecifics when aggregating for diapause.

This study aimed to expand knowledge of the morphological structure of *G*. *italicum*’s sensory organs in the antennal segments. Therefore, an attempt was made to classify different types of olfactory sensilla concerning external morphological features, distribution patterns, and internal receptor structure using scanning electron microscopy and transmission electron microscopy to provide a firm foundation for comparison with other pentatomid species. The investigation also included a detailed morphology and arrangement of thermo-/hygrosensitive and mechanosensitive sensilla assessment. We hypothesize that *G*. *italicum* may possess specific sensilla because the demand for extreme sensitivity in pheromone communication could support the evolution of long chemosensilla due to their higher efficiency in capturing odor molecules. Therefore, we present the results of a morphological and ultrastructural study of a sensory receptor array and interpret the details of these structures within the context of the functional optimality of the sensilla system of this insect.

## 2. Materials and Methods

The study is based on specimens of *G*. *italicum* collected from plants (Apiaceae Lindl.) in Poland (Upper Silesia).

Scanning electron microscopy (SEM): The dry specimens of both sexes (3+3) were dissected to obtain antennae and short-cleaned in water with detergent using an ultrasonic cleaner. Then, a dehydration procedure was applied through a series of ethanol solutions of 50%, 70%, 80%, and 90% for ten minutes each, followed by dehydration with 99.8% ethanol for 20 min twice. Afterward, the antennae were dried at room temperature and were glued with carbon adhesive discs on the pin stubs, which then were coated with a layer of gold (30 nm) using a Q150T ES sputter coater (Quorum Technologies Ltd., Laughton, UK). SEM micrographs were obtained using a Phenom XL (Phenom-World B.V., Eindhoven, The Netherlands) at 15 kV accelerating voltage, with a Back Scatter Detector (BSD) and a field emission scanning electron microscope Hitachi UHR FE-SEMSU8010 (Hitachi High Technologies Corporation, Tokyo, Japan) with a secondary-electron detector (ESD) at 10 kV accelerating voltage.

Preparation of Samples for Transmission Electron Microscopy: The antennae of *G*. *italicum* were cut from the head; the three flagella were divided into two small pieces each and then separately fixed in 2.5% glutaraldehyde prepared in 0.1 M sodium phosphate buffer (pH 7.4, 4 °C, 24 h). After fixation, the material was washed in phosphate buffer (3 × 30 min, at room temperature (RT)), postfixed in 2% osmium tetroxide (2 h at RT), and then washed three times by phosphate buffer for 10 min at RT. The material was dehydrated in the series of ethanol (30, 50, 70, 90, 96, and 100% for 10 min, 10 min, 15 min; 15 min, 15 min, and 4 × 15 min, respectively, at RT), a mixture of 100% ethanol and acetone (1:1, 15 min), acetone (2 × 15 min), incubated in a solution of acetone and epoxy resin (1:1, 1.5 h), and then embedded in epoxy resin (Epoxy Embedding Medium Kit, Sigma, Darmstadt, Germany). The pieces of the flagellum were oriented to a longitudinal section in boxes with epoxy resin (taking into account the arrangement of the sensilla in previous observations with an SEM (scanning electron microscope). The material was cut into ultrathin (50 nm) sections on a Leica EM UC7 RT ultramicrotome (Leica Microsystems, Frankfurt, Germany). The ultrathin sections were mounted on formvar-covered copper grids, stained with uranyl acetate and lead citrate, and analyzed using a Hitachi H500 transmission electron microscope (Hitachi High Technologies Corporation, Tokyo, Japan at 75 kV. All images were taken at a resolution of 1024 × 1024 pixels and saved as TIFF files at the Faculty of Natural Science, TEM (Tokyo, Japan) laboratory of the University of Silesia in Katowice.

Terminology and classification of the sensilla. The morphological identification of the sensilla and the analysis of their features conducted in the present study were based on Altner and Prillinger [28], Li et al. [36], and Shields [39]. Sensilla classification is based on the presence or absence of pores, grooved or smooth surfaces, and whether they possess flexible or inflexible sockets at the base of the sensilla. The ultrastructures of the receptors were compared with the data from Hartenstein [24], Keil [25], Keil and Steinbrecht [26], and Steinbrecht [40] regarding the number of dendrites and their destination. The sensilla’s pores system recognition was additionally based on the longitudinal or ultra-section of the stem sensilla.

## 3. Results

**Figure 1:**
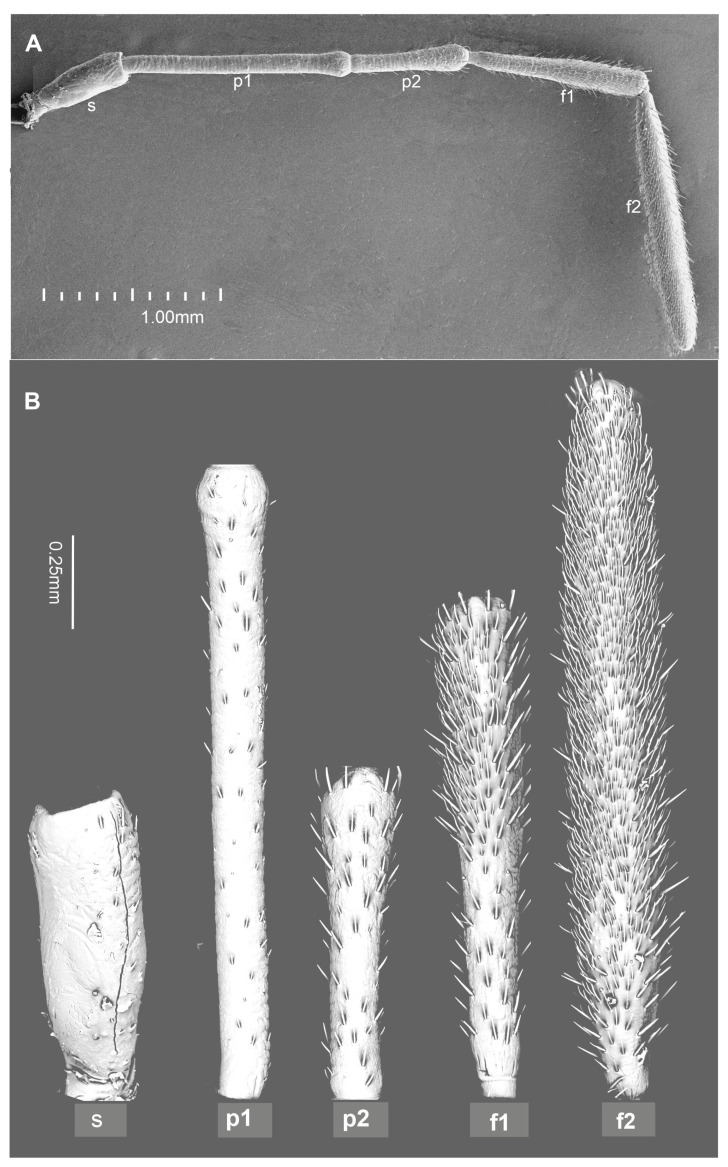
The shape of the antennae (**A**) and individual antennomeres (**B**) of *G*. *italicum*: scape (s), pedicel (p1), pedicel (p2), basifagellum (f1), distifagellum (f2).

The male and female antennae of *G*. *italicum* exhibited the same morphological arrangement and pattern of sensory structures, with no significant difference in the length of their antennomeres (4.99 mm in males and 5.05 mm in females). Generally, the antennomeres varied in shape and size: the scape was shorter (approximately 0.6 mm) and broader than the pedicel. The female’s first pedicel measured 1.25 mm in length, the second pedicel measured 0.7 mm, and the lengths of the f1 (basiflagellum) and f2 (distiflagellum) were 1.0 mm and 1.5 mm, respectively (Figure 1).

Due to the lack of significant sexual dimorphism in antennomeres and sensilla equipment, the results do not differentiate between the sexes of this species.

**Figure 2:**
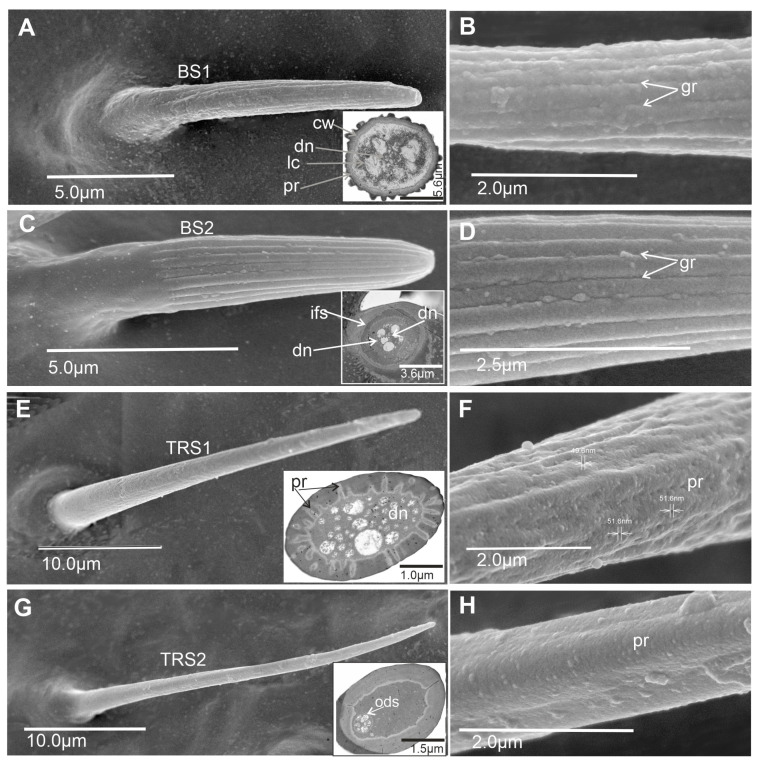
Types of chemosensilla in *G*. *italicum*: (**A**,**B**) Grooved and multiporous wall of the long basiconic sensillum (BS1) with the ultrastructures of the basiconic wall; the pores and at least seven dendrites are visible. (**C**,**D**) Deeply grooved and porous wall of the short basiconic sensillum (Bs2) and cross-section at the base of the sensillum with numerous dendrites. (**E**,**F**) Shorter trichoid sensillum (TRS1) with a porous stem but no grooved wall, numerous dendrites are visible in the cross-section of the sensillum. (**G**,**H**) Long and narrow trichoid sensillum with slightly visible pores (TRS2) and cross-section near the base of TRS2; six dendrites are visible in the dendritic sheath. Abbreviations: cw, cuticular wall with several pores; dn, dendrites; gr, grooved and porous wall; ifs, inflexible sockets; lc, lymph cavity; ods, outer dendritic sheath.

**Figure 3:**
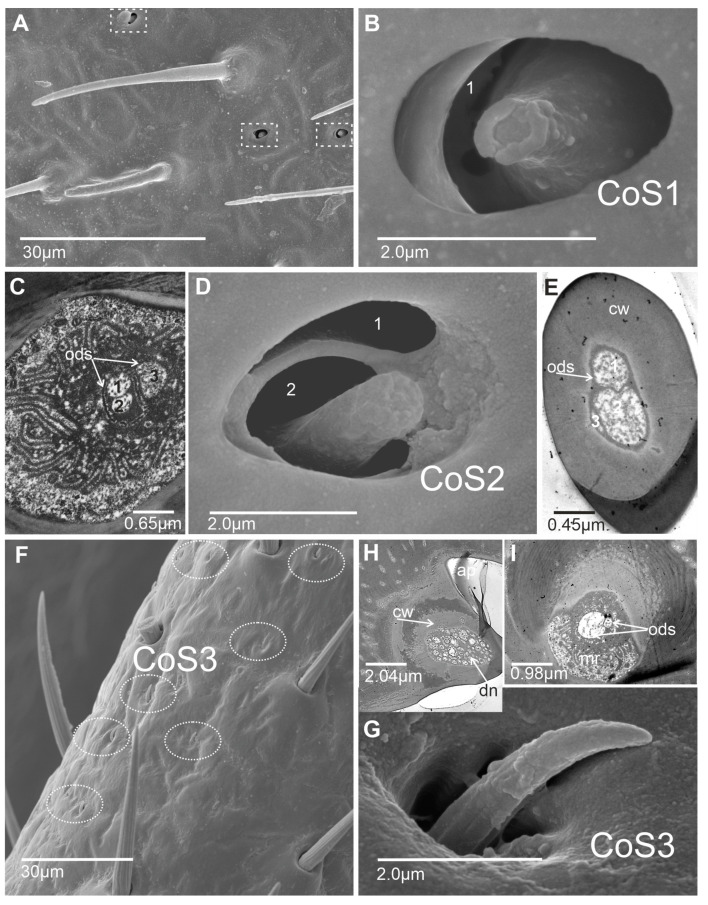
Types and distribution of the coeloconic sensilla (CoS 1–3): (**A**) Coeloconic sensillum (marked as a square) was observed on the basiflagellum (f1). (**B**,**C**) Coeloconic sensillum (CoS1) and cross-section bellow the cuticular surface with the three dendrites ((1+2)+3)). (**D**,**E**) Coeloconic sensillum (CoS2) with two chambers (1, 2) and a cross-section of the wall peg showing three dendrites ((1+2)+3)). (**F**) Distribution of the coeloconic sensilla (CoS3) on the flagellomere (f1) (oval dotted lines). (**G**) The wall grooved of the CoS3. (**H**) The sagittal section of the wall and base of the sensillum (CoS3) showing a porous wall and numerous dendrites. (**I**) Cross-section at the base of the peg with the microvilli (mr) in the lymph cavity of the tormogen cell and the bundle of dendrites (dn). Abbreviations: ap, aperture of holl; cw, cuticular wall with several pores; dn, dendrites; gr, grooved and porous wall; ods, outer dendritic sheath.

**Figure 4:**
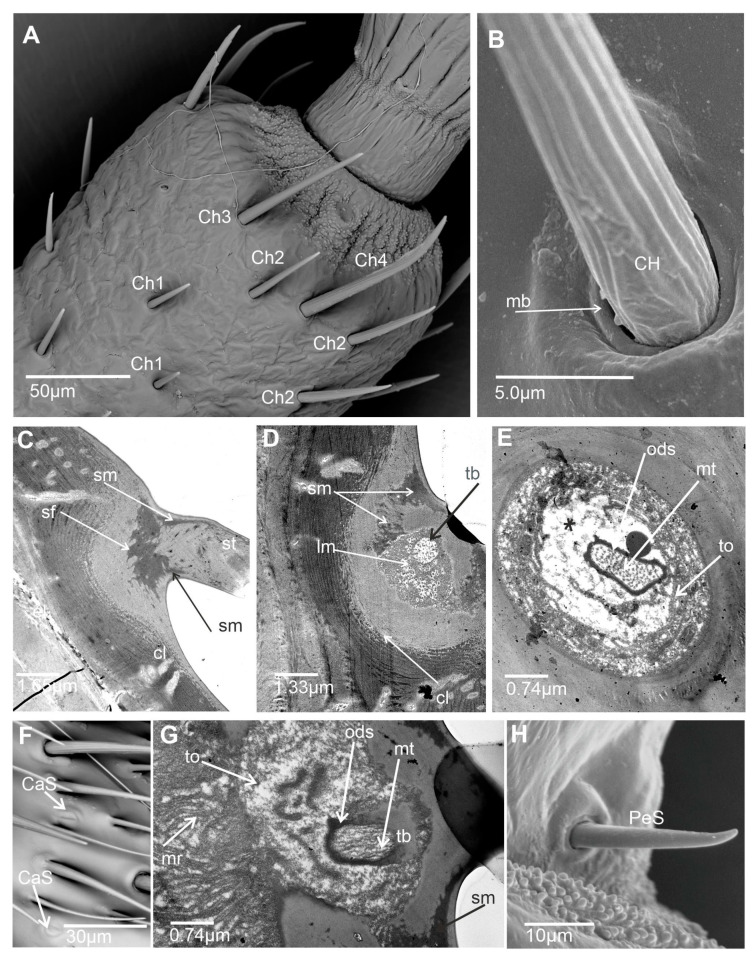
Types and ultrastructure of the mechanosensilla: (**A**) Subtypes of chaetic mechanosensilla according to their length (Ch1–Ch4). (**B**) Flexible socket with the visible external membrane (mb) and the thin grooved surface of the mechanosensillum stem. (**C**) Longitudinal ultra-section at the base of the mechanosensillum showing the shape of the socket membrane (sm) and suspension fibers. (**D**) Deeper ultra-section of the mechanosensillum showing the tubular body (TB) and lymph cavity (lm). (**E**) Tubular body in the ultra-cross-section at the base of the chaetic sensillum. (**F**) Shape of the campaniform sensillum (CaS). (**G**) Ultrastructure of the tubular body (tb) of campaniform sensillum. (**H**) Peg sensillum (PeS) located on the edge of the adjacent antennomeres. Abbreviations: cl, cuticular layer; el, epidermal layer; ods, outer dendrite sheath; sf, suspension fiber; mt, microtubules; to, tormogen cell, marked as a black start; lm, lymph cavity; mb, external membrane of the socket; sm, socket membrane; st, stem of the sensillum.

Six main types of sensilla were recognized: basiconic, trichoid, coeloconic, chaetic, campaniform, and peg sensilla. Morphologically, sensilla were divided into subtypes based on their shape and length. The presented ultrastructure of the receptors supported their possible functions. Consequently, two subtypes of basiconic sensilla (BS1 and BS2), two subtypes of trichoid sensilla (TRS1 and TRS2), three subtypes of coeloconic sensilla (CoS1, CoS2, and CoS3), four subtypes of chaetic sensilla (Ch1, Ch2, Ch3, and Ch4), and one type each of campaniform sensillum (CaS) and peg sensillum (PeS) were recognized (Figure 2, Figure 3 and Figure 4).

Ultrastructure dendritic elements of the receptors, the pore systems of the wall of the sensillum, and the inflexible/flexible sockets revealed the three primary functions of the sensilla: olfactory, thermo–hygroreceptive, and mechanoreceptive. The main characteristics of the dendrites’ ultrastructure are represented by the sensillum base below the cuticular surface as well as the stem sensillum protruding above the cuticular antennal surface.

### 3.1. Categories of the Sensilla

#### 3.1.1. Olfactory Sensilla

**Figure 5:**
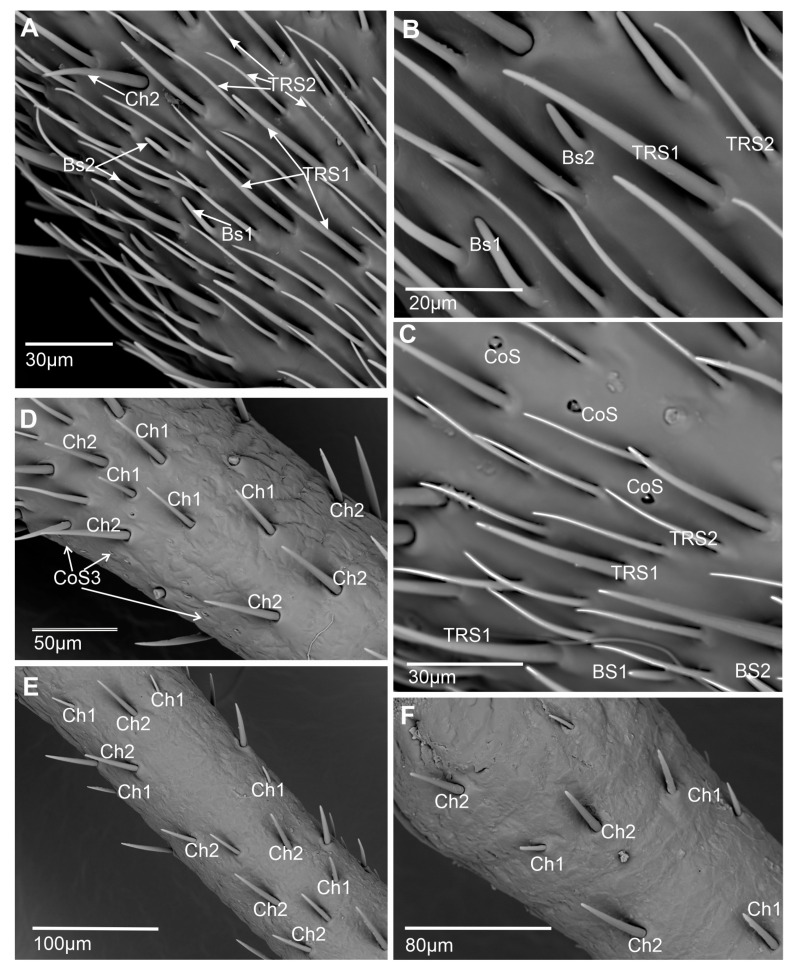
Types and distribution of the sensilla: (**A**,**B**) Sensilla densely distributed on the last flagellomere, with chemosensilla (TRS1 and TRS2, BS1 and BS2) firmly covering the surface, coeloconic sensilla (CoS1-2) are less numerous. Ch3 and Ch4 are less numerous than chemosensilla. (**C**,**D**) The second flagellomere is in proximal parts, where all types of sensilla are densely distributed. (**E**) The first flagellomere, where Ch1 and Ch2 are mainly present. (**F**) The pedicel, where Ch1 and Ch2 are rarely distributed.

- Basiconic sensillum (BS1) is scattered on the first and second flagellomeres and frequently observed in the imaging area (Figure 5A–C). This cone-like sensillum is grooved and has a porous wall. The smooth proximal part is embedded in an inflexible socket (Figure 2A,B), and this sensillum is recognized as longer (L = 11.2–14.2 µm) than BS2 (Table 1). The sensillum’s stem is wide, stiff, and rounded at the end. Additionally, the sensillum ultra-section indicated groups of dendrites in the lymph cavity (Lc) inside the sensillum, confirming its olfactory function.
- Basiconic sensillum (BS2) is distributed randomly in the first and second flagellomeres, with only several such sensillum observed (Figure 5A–C). The sensillum’s stem is wide, stiff, and rounded at the end, and it is embedded in inflexible sockets on the cuticle surface. BS2 is recognized as a short sensillum (L = 7.4–8.41 µm) with a grooved and multiporous wall (Figure 2C,D). These structures are present on the non-proximal area of the sensilla’s cuticle (Figure 2C). The ultra-section at the base of the sensillum indicated numerous dendrites (dn) (about 37) and documented its olfactory function.
- Trichoid sensillum (TRS1) has a round base and a long cylindrical cuticular multiporous shaft tapered apically into a sharp tip (Figure 2E,F). It is classified as a shorter sensillum (Table 1) than TRS2. Pores about 50 nm in diameter are densely distributed along the entire length of the sensillum. The base is embedded in an inflexible socket. The cross-section shows pores in the wall and numerous dendrites inside the lumen cavity of the sensillum, confirming its olfactory function. This sensillum is numerous on the first and second flagellomeres (Figure 5A–C).
- Trichoid sensillum (TRS2) has a round base and a long, thin, cylindrical cuticular multiporous shaft tapered apically into a sharp tip (Figure 2G,H). It is classified as a longer sensillum (Table 1) than TRS1. The base is embedded in an inflexible socket. The cross-section shows a few pores on the wall and several dendrites (at least five). This sensillum is numerous and distributed throughout the first and second flagellomeres (Figure 5A–C).

#### 3.1.2. Thermo–Hygroreceptive Sensilla

- Coeloconic sensillum (CoS1) has only a few irregular cavities in several numbers (Figure 3A) in the middle and distal flagellum in both sexes. The short peg-like sensillum is embedded in an inflexible socket in a shallow, oval singular cavity (Figure 3B). The proximal part is wider than the distal, with a narrow, rounded end. The wall pores are invisible, so this sensillum is treated as non-porous. We could only identify a slightly invaginated pore (molting pore) at its apical tip. The cross-section below the cuticle surface shows the presence of the three dendrites; however, two dendrites (no. 1 and 2) are surrounded by an outer dendritic sheath (ods), similar to the third dendrite (no. 3) (Figure 3C). The composition of the three dendrites is visible in the cross-section at the base of the sensillum. Additionally, the microvilli (mr) are also observed. The number and arrangement of the three dendrites can suggest a thermo–hygroreceptive function of the sensillum.
- Coeloconic sensillum (CoS2) is present in several numbers in the middle and distal flagellums (Figure 3D). The short peg-like sensillum is probably embedded in an inflexible socket in a shallow cavity of two chambers (Figure 3D). The proximal part is perhaps broader than the distal, with a blunt end and slight protrusion. The surface of the peg is smooth and shows no indications of wall pores except the slightly invaginated molting pore at the apical tip (Figure 3E). The ultra-section at the base of the peg shows the presence of the three dendrites; however, two dendrites (no. 1 and 2) (Figure 3E) are surrounded separately by a dendritic sheath from the third dendrite (no. 3). Dendrite no. 3 probably terminates at the base of the sensillum, while the other two dendrites probably extend into the lumen of the peg to its distal end. The single dendrite at the base is likely responsible for thermoreception, while the other two dendrites are responsible for hygroreception.
- Coeloconic sensillum (CoS3) is a cone-like sensillum with a profoundly grooved, porous wall. The base of the sensillum is probably embedded in an inflexible socket in a deeper and narrower cavity than CoS1 and CoS2 (Figure 3G). The stem slightly protrudes from the cavity. Numerous sensilla of this type were observed on the lateral side of the basiflagellum (Figure 3F). The ultrastructure of the peg shows the presence of multiple dendrites (Figure 3H,I), suggesting their olfactory function.

#### 3.1.3. Mechanoreceptive Sensilla

- Chaetic sensillum (Ch) belongs to the group of mechanosensilla, primarily distinguished by their length (Ch1 = 14.3–20.5; Ch2 = 28.4–36.4; Ch3 = 50.2–60.0; Ch4 = 66.5–73.0) (Figure 4A) (Table 1). These sensilla are stout bristles connected to the cuticular surface by a socket equipped with a flexible external membrane (mb), allowing possible deformations of the sensilla (Figure 4B). Mechanoreceptive chaetic sensilla are straight in shape, broad at the basal part, and narrow at the distal part. The external surface of the stem is grooved, but the pattern differs from the wall grooves of the chemosensilla. These sensilla are positioned on the antennal surface at a larger angle (about 45°) than basiconic and trichoid sensilla, making them visible as they stick out (Figure 1B and Figure 4A).

In longitudinal ultra-sections, the position and structural components of the socket, such as socket membrane (sm), suspension fibers (sf), the inner structures (thickness of the cuticular layer (cl) and epidermal layer (el)) (Figure 4C,D) are visible. The ultra-section at the base of the chaetic sensillum shows a tubular body (tb) with a large outer dendritic sheath (ods) and numerous microtubules (mt) (Figure 4E). Outside the dendrite sheath, the tormogen cell (To) with microvilli increases in diameter near the socket (Figure 4D,E). Dendrites are absent inside the sensillum stem (Figure 4C), indicating only a mechanosensitive function.

The distribution of sensilla varies across particular antennomeres (Figure 1B and Figure 5A–F). The scapus (s) and pedicel (p) mainly possess the chaetic sensilla Ch1 and Ch2 types, which are rarely present on these antennomeres. On the basiflagellum (f1), Ch1 and Ch2 are dominant sensilla types; a similar arrangement is found on the distiflagellum (f2). In the distal area of the f1, several chaetic sensilla (Ch3 and Ch4) are indicated. On both members of the flagellum, among densely spread chemo sensilla, several chaetic mechanosensillas were observed, mainly Ch3 and Ch4.

- Campaniform sensillum (CaS) is a dome and oval-shaped structure with a single pore in the middle (Figure 4F) and embedded in sockets with a flexible membrane (sm) (Figure 4G). The campaniform sensillum has an ultrastructure similar to the chaetic sensillum. Dendrites in the distal outer segment are encased by a dendrite sheath (ods) and form a tubular body (tb) (cytoskeletal complex structure) consisting of multiple tiny, tightly packed microtubules (Figure 4G). The tormogen (to) cell forms the large lymph cavity and numerous microvilli (mr), and the tubular body (tb) attaches to the center of the cap and terminates at its base. Several sensilla (2–4) are located in different places in each antennomere, functioning as proprioceptors responding to strains in the exoskeleton.
- Peg sensillum (PeS) is the conical-shaped stiff sensillum with a non-porous wall but a flexible socket, categorized as the proprioceptors that occur in the proximal section of the pedicel and are directed to control the position of the scapus (Figure 4H).

## 4. Discussion

In the present study of *G*. *italicum,* no sexual dimorphism was observed regarding the shape and size of the antennae or the antennal sensory organs was observed, similar to findings in other pentatomomorphan species such as *Leptoglossus occidentalis* and *L*. *zonatus* [10], *N*. *viridula* [31], *A*. *chinensis* [34], *Perillus bioculatus*, *Dolycoris indicus*, *Plautia crossota* [35], *H*. *halys* [37], and *Riptortus pedestris* [41]. Similar sets of sensilla have been recorded among the studied species of pentatomomorphan bugs, including basiconic, trichoid, coeloconic, chaetic, peg, and campaniform sensilla, with several combinations of the subtypes.

The sensory organs in *G*. *italicum* (Pentatomidae: Podopinae) do not show significant differences in the general pattern of the sensilla when compared to other pentatomid species. However, the present study’s analysis of the 13 sensilla subtypes (Table 1) primarily focuses on detailed external micromorphology and dendrites’ ultrastructure in order to recognize the exact function of the particular sensilla types.

### 4.1. Morphology and Ultrastructure of Basiconic and Trichoid Sensilla

Insects perceive olfactory stimuli using olfactory receptors (OR) or ionotropic receptors (IR) and, in some cases, gustatory receptors (GR), localized in dendrites sensitive to odor molecules [42]. The organization and function of the olfactory system have been studied in many insects, resulting in a wealth of information from multiple species [43]. Olfaction in insects begins when a volatile compound diffuses into porous structures of sensilla scattered mainly across the antennae [44,45]. The responses of the insects to odors vary in their dynamics. Each sensillum houses one or more olfactory sensory neurons (OSNs). In different sensilla types, olfactory sensory neurons (OSNs) typically respond to different odors. Each sensillum belonging to a given class might house one OSN expressing receptor X and another expressing receptor Y. For example, in *D. melanogaster,* IRs are a functional receptor type of OSNs in double-walled coeloconic sensilla, and ORs are mainly expressed in OSNs located in single-walled basiconic and trichoid sensilla [46,47]. If the compound is recognized by an olfactory receptor complex in the membrane of one of these OSNs, binding may trigger the neuron to fire, sending a signal to the brain.

A greater number of chemoreceptors increases the potential for perception of the chemical environment. It has been noted that the high number of sensilla found on the antennal flagellum of Heteroptera may be used to detect olfactory cues during orientation at a long distance from a host plant. These insects likely detect volatiles that emanate from plant surfaces and interact with chemicals and textures during exploration and antennation of the plant’s surface [48]. Olfactory sensilla show significant diversification in shape and different responses to smell even within the same species, in different species, or between the sexes [49]. Insect olfactory sensilla fall into two fundamentally different categories, the porous single-walled and double-walled wall-pore sensilla in the nomenclature of Altner [50].

In *Graphosoma*, five types of sensilla with olfactory functions were recognized based on the morphology and ultrastructure of the dendrite. These include two subtypes of grooved basiconic porous double-walled sensilla (BS1 and BS2) of two lengths and single-walled trichoid porous sensilla subtypes (TrS1 and TrS2) also of two lengths. Additionally, a coeloconic sensillum has a grooved wall and numerous branched dendrites at the base of the sensillum. Thus, five different olfactory sensilla were found on the same antenna, with the most dominant being a long and slender trichoid sensillum distributed on the basiflagellum and distiflagellum.

The selective pressures leading to this diversification (including numerous long sensilla) are also evident in a few other cases. For instance, the demand for extreme sensitivity in moth pheromone communication has motivated the evolution of long sensilla trichodea, which are highly efficient at capturing odor molecules. Different sensilla of the same morphological type may contain different odorant-binding proteins (OBPs) of the same or different subclasses. However, OBPs of different subclasses are not co-localized in the same individual sensory hair. The presence of a given OBP is more related to the receptor cells’ functional specificity than to the sensillum’s morphological type, suggesting a role for OBPs in stimulus recognition [49].

Pentatomids rely heavily on olfaction for their intra- and interspecific communication through pheromones (aggregation, sexual, and alarm) and kairomones (plant volatiles). Porous basiconic sensilla, described in most studied pentatomids, are the primary receptors involved in volatile perception [37]. Nevertheless, in *H*. *halys*, only trichoid sensilla showed characteristics typical of olfactory function [37], which are identical in morphology with TrS2 in *Graphosoma*. However, the basiconic sensillum SB1 and SB2 in *Graphosoma* are comparable to SB-D and SB-C of *H. halys*; consequently, these sensilla are presumed to possess olfactory roles. In other species, these basiconic sensilla are referred to by different abbreviations but are classified as olfactory sensilla morphologically and functionally. Short basiconic sensilla (SBsh) in four pentatomids [35] are identical to SB1 in *Graphosoma*, in *N*. *viridula* (termed “type 4 sensillum” by Brézot [31] and in *C*. *siccifolia* and *C*. *purpurea* (termed “s.b. II” by Rani and Madhavendra [30]. The porous basiconic sensilla in different taxa of insects can probably perceive long-range chemical stimuli regarding the host’s location and/or sexual recognition [51]. In other taxa of Heteroptera, the olfactory sensilla are also represented by several different types/subtypes. The presence of pores on basiconic and placoid sensilla in some Gerromorpha taxa reflects the antennae’s ability to perceive various chemical stimuli. In *Leptoglossus* (Coreidae), six multiporous sensilla with an inflexible socket (M4 to M9) were indicated as having a typical olfactory function, and additionally, three multiporous sensilla (M1–M3) with flexible socket [10]. This difference in the number of types of olfactory sensilla is notable when compared to other studied Pentatomorpha species. The group of olfactory sensilla BS1, BS2, TrS1, TrS2, and CoS3 in *Graphosoma* are morphologically and functionally similar to sensilla M6, M7, M5, M2, and M8 in *Leptoglossus*. Despite the presence of multiporous sensilla in flexible sockets (M1–M3) in *Leptoglossus*, such sensilla was not confirmed in *Graphosoma*.

The thin-walled long trichoid sensilla and thick, grooved wall multiporous sensilla were identified separately in *Oncopeltus fasciatus* (Dallas, 1852) [52], *Oxycarenus laetus* Kirby, 1891 (Lygaeidae) [53] and *Neomegalotomus parvus* (Westwood) (Alydidae) [54], which is typical for most species. However, in *Graphosoma*, the thin-walled long trichoid sensilla represents two subtypes (TrS1, TrS2), and the thick, grooved wall multiporous sensilla possesses three subtypes (BS1, BS2, CoS3). In *H*. *halys* and other pentatomids, multiporous olfactory sensilla are usually documented in two or three subtypes [29,30,33]. These differences in the sensilla subtypes among heteropteran species could be an adaptation to olfaction related to bugs’ pheromones and the variety of substances they need to detect during the location and selection of the host and non-host plants, all emitting specific volatiles [55,56]. In most insects, the number of grooved double-walled (dw-wp) sensilla studied is still small compared with single-walled (sw-wp) sensilla. So far, no pheromone receptor cells are associated with the double-walled sensillum. Altner et al. [57] compared both sensillum types in *Periplaneta* and showed that dw-wp sensilla were usually sensitive to more polar compounds (e.g., short-chain fatty acids) than sw-wp sensilla, which mainly responded to apolar, long-chain fatty alcohols or esters (included in insect pheromones). The receptors of the sensilla of double-walled (SB1, SB2, and Cos3) in *Graphosoma* probably recognize the various plant odors in contrast to the receptors of the sensilla of single-walled (TrS1, TrS2), which may participate in the recognition of pheromones (aggregation, sexual and alarm). Fundamentally similar bauplan of multiporous double-walled and single-walled types sensilla in insects as different as Orthoptera, Heteroptera, and Diptera and their widespread occurrence in so many insect orders [28] favor the notion that these types have a very old origin they are specific for insects. The two categories of olfactory sensilla in insects do not meet structural equivalents in the other arthropod taxa [58].

### 4.2. Morphology and Ultrastructure of Coeloconic Sensilla

The coeloconic sensilla described on antennae of insects also occurs on the trunk of various larvae [24] and possesses thermo-sensitive neurons often combined with two hygro-sensitive neurons, forming a sensory triad consisting of a moist-sensitive neuron, a dry-sensitive neuron, and a cold-sensitive neuron (MDC-triad), described mainly for single-walled sensilla with a non-porous peg on an inflexible socket [59,60,61,62]. In some species (stick insects, cave beetles), lamellation of the outer dendritic segment refers to cold-sensitive neurons in the triad [63,64]. In contrast, exclusively chemo-sensitive coeloconic sensilla, with lamellated outer dendritic segments of sensory neurons, have been described on Pieris rapae palps [65,66]. According to Ruchty et al. [66], lamellation of the outer dendritic segment of a sensory neuron does not necessarily predict its function as a cold-sensitive neuron. A combination of thermo- and chemo-sensitive neurons may be observed in coeloconic sensilla in several insects [57,67]. Moreover, thermo-sensitive coeloconic sensilla is frequently found in insects. The sensor is double-walled and innervated by three unbranched dendrites, and the neuron responds to changes in air temperature (convective heat), radiant heat, and cold in response to a drop in air temperature [66]. Insects show a variety of temperature-guided behaviors, so specific adaptations of thermosensitive sensilla are expected depending on whether heat reaches the sensillum via air movements or radiant heat.

In the present study, three distinct types of coeloconic sensilla were identified based on external shape and differences in the number of their dendrites’ and their branching pattern. Coeloconic sensilla CoS 1 (in a singular cavity) and Cos2 (with two chambers in the cavity) possessed a smooth wall (not porous and not grooved) and three neurons with unbranched dendrites without lamellations. We suggest that these sensilla represent a triad. Similarly, three unbranched dendrites (DOS) were also described in another type of coeloconic sensillum (II) in the tropical katydid of the genus *Mecopoda* [64]. Nevertheless, the differences in the ultrastructure of the coeloconic receptors are more complex; some taxa, such as the tropical katydid of the genus *Mecopoda* (Orthoptera), have coeloconic sensilla with two dendrites [64]. Hygroreceptors often occur together with thermoreceptors in pegs that have no pore system. The peg-in-pit morphology protects the sensory peg against harsh mechanical contact, such as during antennal grooming, and probably prevents evaporative water loss and cooling [50,68]. Another characteristic is that they occur singularly and rarely compared to all other sensilla types. The several non-porous sensilla (A5) recognized in Coreidae (*L*. *occidentalis*, *L*. *zonatus*) were morphologically and functionally classified as thermo–hygroreceptors [10], and this sensillum corresponds exactly to the CoS1 in *G*. *italicum*. Analysis of the coeloconic sensilla in *Eocanthecona furcellata* (Pentatomidae) and *Graphosoma* shows the same type of CoS1 but reveals a difference in the shape of CoS2 (SCo2 in *E*. *furcellata* was found only in males, and can be compared to the CoS3 presented in *Graphosoma*) [34]. In the mentioned pentatomid taxa (*O*. *nigricornis*, *Nezara viridula* [29], *Cyclopelta siccifolia*, *Chrysocoris purpureus*, *P*. *building*, *Perillus binoculars* [30], and *H*. *halys* [37]), the non-porous coeloconic sensilla embedded in different chambers/cavities with small apertures and present on the flagellum were reported as thermo–hygroreceptors [33,35].

Only the third type of coeloconic sensilla (CoS3) is marked as having an olfactory function different from Cos1 and Cos2 based on the ultrastructure of the peg base showing numerous dendrites. The coeloconic sensilla (M8 and M9) classified as multiporous chemosensilla, identified in *L*. occidentalis and *L*. *zonatus* of the family Coreidae [10], are morphologically similar to the present sensillum CoS3. According to Kim et al. [41], the antennae of adults of *Riptortus pedestris* (Alydidae) showed that Co2 had numerous pores on the surface, indicating a chemosensory function. In some studies, it is known that porous coeloconic sensilla have olfactory functions and may receive chemical stimuli to locate hosts or identify pheromones [15].

### 4.3. Mechanoreception (Exteroceptors and Proprioceptors)

Mechanosensilla spread across the entire body of insects, are the primary organs for detecting mechanical stimuli from the external environment, such as air or water movements, air currents generated by predators, mates, and competitors, and self-movement of body regions in touch [13,25,26]. Mechanosensilla appear in various morphological shapes and lengths on the antennae of many insects, leading to inconsistencies in their comparison among different species. The main shapes of mechanosensilla are trichoid, chaetic, bristle, styloconic, basiconic, and trichobotria, and are primarily composed of exocuticular material developed with the trichogen cell [26,69,70]. Externally, the sensory organ consists of the stem, usually with a grooved or smooth wall, and a moveable socket with an articulated membrane composed of rubber-like protein resilin. The mechanosensillum is innervated by one bipolar neuron, whose unbranched dendrites attach to the outer dendritic sheath, either centrally or laterally, forming a tubular body with varying numbers of microtubules at the base of the sensillum [11,13]. Depending on their distribution, mechanosensilla can function as exteroceptors or proprioceptors [13,24].

In *G*. *italicum*, three types of mechanosensitive sensilla (chaetica, peg, and campaniform) were identified. The most abundant chaetic sensilla (subtypes Ch1–Ch4) are distributed differently across each antennomere. They are also commonly found on the antennae of many other insects [71,72]. Morphological and ultrastructural analysis of the sensory cells in this species indicates their mechanoreceptive function. Typical of such a sensilla is a flexible socket and a large tubular body with a parallel arrangement of microtubules associated with electron-dense material terminating in the proximal part of the sensilla. The tubular bodies, probably of all subtypes of chaetic sensilla of *G*. *italicum*, have a complex structure, indicating their sensitivity. Variations in the amount and arrangement of electron-dense material at the tip of the tubular bodies likely reflect differences in viscoelastic properties related to the functional characteristics connected to the size of the sensilla subtypes. According to data on the function of the mechanosensilla, the position of the tubular body at the base of the sensillum stem, and the number of microtubules vary in some insect species [73].

In *Graphosoma*, chaetic sensilla with different lengths (Ch1–Ch4) were observed. In all subtypes, their stiff, grooved stem is more perpendicularly positioned in the flexible sockets relative to the antennal segment surface. They significantly stand out from other hair-like or basiconic sensilla, which are situated more horizontally along the long axis of the antennal segments. Long chaetic sensilla are adapted for receiving tactile stimuli, air currents, substrate vibrations, and shocks detected by sensilla from exploratory movements of the antennae. Chaetic sensilla in most pentatomid species are documented as a single type with longitudinal grooves located in an open articulating socket in *A*. *chinensis* [34]. The subtype Ch1 is present in five species (*Cressona divaricata*, *Eurydema dominulus*, *Halyomorpha halys*, *Plautia crossota*, and *Scotinophara lurida*), the second type Ch2 only in *Eocanthecona furcellata* (with spoon-shaped tips detected only on the antennal scape and basal pedicel) [36], and the bristle-like type 3 in *N*. *viridula* [31]. These can be compared with Ch4 or Ch3 in *Graphosoma*. In other taxa, the aporous sensilla, with flexible socket types A2 and A3 in *Lepotglossus* [10], corresponds to Ch2/Ch3 and Ch4 in *Graphosoma*.

The longer and narrower chaetic sensilla (Ch4) in *Graphosoma* project from the distal margin of the second pedicel and basisflagellum. They probably also provide information about the relative positions of the antennal segments because a typical external proprioception sensillum (PeS) is present on the ventral side between the pedicel and the scapus. The connection between particular antennomeres (p2, f1, f,2) is based on flexible insertion structures (annuli) whose position is likely controlled by the mentioned chaetic sensilla. Such organization of the proprioception between the scapus and pedicel was found in other species, such as *Leptoglossus* [10], and described as basiconic sensilla with a flexible socket (A1) similar to PeS in *Graphosoma*. Proprioceptive sensilla are widespread in insects across different body areas [11] and are stimulated by mechanical factors caused by body part movements [56,74,75,76].

Campaniform sensilla in insects are dome and oval-shaped structures innervated by one mechanosensitive bipolar neuron, performing proprioception functions [11,28,77,78]. Ultrastructural analysis of campaniform sensilla in *G*. *italicum* found on the flagellum confirms the presence and organization of the dendrite, terminating at the base of the dome and forming a tubular body. The sensillum is responsible for proprioception. In many heteropteran species, a group of dome-shaped aporous sensilla localized proximally on the ventral side of the scapus corresponds to the morphological characteristics of campaniform sensilla [9,10,35,75]. Across other insects (e.g., stick insect, drosophila, and cockroaches), campaniform sensilla usually possess a similar shape and function, although their size may vary among species [33,77,78,79].

## 5. Conclusions

In *G*. *italicum*, various types of antennal sensilla were morphologically studied, and sensilla patterns were compared to other Pentatomidae. Olfactory sensilla in *Graphosoma* are represented by five morphological forms, whereas two to four types of olfactory sensilla are observed in other pentatomids. The ultrastructure of the long and thin trichoid sensillum (TrS2) is documented with six dendrites and several pores in the wall, indicating a limited or specific function in olfaction. Previous studies identified such trichoid sensilla as mechanosensilla or microtrichia (non-sensory structures). The variety of chemosensitive sensilla on the *Graphosoma* antennae indicates that various chemical information might be necessary for host recognition and acceptance.

The basiconic or coeloconic sensilla with longitudinal grooves (double-walled) and pores deeply located between grooves allow odors to pass through. Sensilla with this type of morphology have been conserved through hundreds of millions of years of insect evolution. They can be found in many and perhaps all insect orders, suggesting a critical function in chemosensory coding.

Despite the two morphological types of coeloconic sensilla, the ultrastructure of the receptors shows identical arrangements of the three dendrites. The number and distribution of thermo–hygroreceptors may indicate a general function of these sensilla, such as thermoregulation and maintaining a stable water balance.

The mechanosensilla set is typical for a pentatomid pattern and consists of several campaniform sensilla, one peg proprioception sensillum, and numerous chaetic sensilla of different lengths (Ch1–Ch4).

## Figures and Tables

**Table 1:** Morphological characteristics and distribution sensilla of *G*. *italicum* (N = 12).

Type of Sensillum	Subtypes of Sensilla	Range of Length (µm)	Wall	Tip	Socket	Distribution
Basiconic sensilla	BS1 BS2	11.2–14.2 7.4–8.4	Groves and porous Deeply grooves and porous	Rounded	Inflexible	Middle and distiflagellum
Trichoid sensilla	TRS1 TRS2	27.0–29.1 30.0–33.2	Porous Porous	Acute	Inflexible	Middle and distiflagellum
Coeloconic sensilla	CoS1 CoS2 CoS3	- - -	No porous No porous Groves and porous	Blunted	Inflexible	Middle and distiflagellum
Chaetic sensilla	Ch1 Ch2 Ch3 Ch4	14.3–20.5 28.4–36.4 50.2–60.0 66.5–73.0	Groves and no porous	Sharp	Flexible	On each antennomeres, but Ch3 and Ch4 are more numerous on the two last antennomeres
Campaniform sensillum	CaS		Molting pore	Cupola	Flexible	Several on each antennomeres
Peg sensillum	PeS		No porous	Rounded	Flexible	Proximal edge of the pedicel

## Data Availability

The original contributions presented in the study are included in the article, further inquiries can be directed to the corresponding author.
